# A nomogram for predicting cancer-specific survival in patients with osteosarcoma as secondary malignancy

**DOI:** 10.1038/s41598-020-69740-2

**Published:** 2020-07-30

**Authors:** Yanqi He, Han Liu, Shuai Wang, Jianjun Zhang

**Affiliations:** 1 Department of Respiratory and Critical Care Medicine, West China Hospital, Sichuan University, Chengdu, China; 2 Department of Respiratory Medicine, The First Hospital of Jilin University, Changchun, China; 3 Department of Vascular Surgery, The First Hospital of Jilin University, Changchun, China; 4 Department of Oncology, Shanghai Jiao Tong University Affiliated Sixth People’s Hospital, Shanghai, China

**Keywords:** Cancer, Bone cancer

## Abstract

The prognostic factors for survival among patients with secondary osteosarcoma remain unclear. The aim of this study was to develop a practical nomogram for predicting cancer-specific survival (CSS) in patients with osteosarcoma as a secondary malignancy. The surveillance, epidemiology, and end results database was used for the identification of osteosarcoma cases. The total sample comprised 5860 cases of primary osteosarcoma and 268 cases of secondary osteosarcoma during the period from 1973 to 2015. The CSS and overall survival (OS) of primary and secondary osteosarcomas were analyzed. The predictors of CSS for secondary osteosarcoma were identified and integrated to build a nomogram. Validation of the nomogram was performed using concordance index (C-index) and calibration plots. The results indicated that patients with secondary osteosarcoma had poorer CSS and OS than patients with primary osteosarcoma. The nomogram model exhibited high discriminative accuracy in the training cohort (C-index = 0.826), which was confirmed in the internal validation cohort (C-index = 0.791). In addition, the calibration plots confirmed good concordance for prediction of CSS at 3, 5, and 10 years. In conclusion, we developed a practical nomogram that provided individual predictions of CSS for patients with secondary osteosarcoma. This nomogram may help clinicians with prognostic evaluations and with the development of individualized therapies for this aggressive disease.

## Introduction

Osteosarcoma is the most common primary bone malignancy, with an age-standardized incidence rate of 2.9 per 1 million amongst men and 2.2 per 1 million amongst women. Nearly 90% of cases are classified as high-grade osteosarcoma at the time of diagnosis. Osteosarcoma is the most common primary malignant bone tumor among people of all ages^1^. Osteosarcoma may present as a primary malignancy or as a secondary malignancy following other primary malignancies. Secondary osteosarcomas frequently occur due to a genetic predisposition and/or as the consequence of prior cancer therapies^2^. Osteosarcoma is one of the most common secondary malignancies among patients with retinoblastoma, with cumulative incidence of 7% at 20 years of age. Patients often have a large number of mutations in the retinoblastoma susceptibility gene, *RB1*^3,4^. Osteosarcoma is also a common secondary malignancy in childhood cancer survivors. The condition often arises as a result of exposure to radiotherapy and chemotherapy^5–7^. Increased incidences of osteosarcoma are associated with Ewing’s sarcoma and Paget’s bone disease^2,8^.

The survival of patients with osteosarcoma has improved considerably since the 1980s with the advent of multiagent chemotherapy, with overall survival of roughly 20% in metastatic patients and 70% in non-metastatic patients^9,10^. If patients with poor survival can be identified preoperatively, personalized treatment plans may be helpful in decision making. Therefore, there is a critical need to identify the patients who are more likely to experience poor survival and thus benefit from additional therapy. Generally, tumor site, tumor size, patient age, location of metastases, response to chemotherapy, and type of surgery are significant prognostic factors for patients with primary osteosarcoma^11–13^. However, because patients with secondary osteosarcoma generally have a history of prior malignances, this history may affect the speed of diagnosis, treatment intensity, and, eventually, the prognosis of secondary osteosarcoma^14^. Secondary osteosarcoma is rarer than primary osteosarcoma, as published by many authors. Previous studies were limited to case reports and small series^4,5,14–17^. Therefore, the prognostic factors of survival for secondary osteosarcoma remain poorly understood.

Nomograms have been successfully used as prognostic tools for predicting the probability of disease outcomes with a simple visualization figure that integrates the relevant variables in complex mathematical models^18,19^. Nomograms can improve the discriminatory accuracy of outcome predictions; these have therefore been widely used to quantify the risk of various malignancies^20,21^. However, no nomogram has been developed for patients with secondary osteosarcoma to date. The present study developed an elaborate nomogram for assessing individualized prognoses for secondary osteosarcoma in terms of 3-year, 5-year, and 10-year cancer-specific survival (CSS) using data from the Surveillance, Epidemiology, and End Results (SEER) database^22^.

## Methods

### Patients and selection criteria

We queried nine population-based cancer registries in the SEER program to obtain records for patients seen during the period from 1973 to 2015 (November 2017 submission) using SEER*Stat software (version 8.3.5)^23^. The SEER program is a population-based cancer registry system with data collected from 18 registries in 14 states across the U.S., representing nearly 30% of the U.S. population. The selection of osteosarcoma cases was done using the Histologic International Classification of Diseases (ICD)-O-3 codes 9180/3–9186/3, 9192/3–9194/3, and 9120/3. No written informed consent was obtained for this study because the data were de-identified and publicly available.

Patients were divided and classified by their sequence numbers: patients with primary osteosarcoma without any prior malignancy were assigned sequence number = 1, and patients with subsequent osteosarcoma following prior malignancies were assigned sequence numbers ≥ 2. Osteosarcomas that occurred following the primary malignancy were considered as “secondary osteosarcoma” in our study.

The exclusion criteria were missing or incomplete data including survival status and time, age, sex, race, and prior malignancies and diagnosis of osteosarcoma at the time of autopsy or on the death certificate. The demographic and clinico-pathological data of all eligible cases were collected and analyzed.

### Endpoint definition

Cancer-specific death was taken as the primary endpoint of the study. The cause of death was defined as death from osteosarcoma, according to the SEER database. The primary endpoint in this study was defined as the interval between the diagnosis of osteosarcoma and the occurrence of cancer-specific death. The secondary endpoint was overall survival (OS), which was defined as the interval between the diagnosis of osteosarcoma and death from any cause or last follow-up.

### Statistical analyses

Statistical analyses were performed using R software version 3.5.1 (R Foundation for Statistical Computing, Vienna, Austria) and SPSS version 20.0 (IBM Corporation, Armonk, NY). The *t*-test was used to examine differences between mean values. The χ^2^ or Fisher’s exact test was used to compare proportions. Survival was assessed using the Kaplan–Meier method and compared using the log-rank test. Cox proportional hazard regression analyses were performed to identify independent prognostic factors of survival (univariate and multivariate). Significant variables (*P* < 0.1) in univariate analyses were included in multivariate regression analyses. Variables that were significant in multivariable analyses were incorporated to formulate the nomogram.

Adequate discrimination and calibration were performed to test and validate the prognostic accuracy of the nomogram model^24^. Discrimination was quantified using Harrell’s concordance index (C-index), in which an absolute value close to 1 indicates that a nomogram model has strong predictive ability. The nomogram was further subjected to bootstrapping validation (1000 bootstrap replicates) to calculate the relatively corrected C-index. Calibration plots were developed to evaluate predictive accuracy and, further, to assess the concordance between predicted and observed ongoing survival probabilities. A two-sided *P* < 0.05 was taken to indicate statistical significance.

## Results

### Study cohorts

The total sample was comprised of 6128 patients, out of which 5860 patients were diagnosed with primary osteosarcoma (sequence number = 1) and 268 patients were diagnosed with secondary osteosarcoma (sequence number ≥ 2). Osteosarcoma was a second malignancy in 231 cases, third malignancy in 34 cases, fourth malignancy in 2 cases, and sixth malignancy in 1 case. Comparisons of baseline demographic and clinicopathological characteristics between patients with primary and secondary osteosarcomas are presented in Table 1. Patients with secondary osteosarcoma were older than those with primary osteosarcoma at the time of diagnosis (55.1 vs. 29.8 years, respectively; *P* < 0.001), 194 (72.4%) secondary osteosarcoma patients were older than 40 years at diagnosis. The ratio of females to males was higher in the secondary osteosarcoma group than in the primary osteosarcoma group (53.4% vs. 44.8%, respectively; *P* = 0.007). Furthermore, the primary site was less likely to be an extremity in cases of secondary osteosarcoma, the pelvis was the most commonly affected site (77 out of 268, 28.7%). Non-pagetic osteosarcoma was more common in patients with primary osteosarcoma, while pagetic osteosarcoma was more common in patients with secondary osteosarcoma, which demonstrates the significant differences in histological subtype between groups. In cases of secondary osteosarcoma, the first primary malignancies included 157 carcinomas (58.6%), 42 sarcomas (15.7%), 41 lymphomas/leukemias (15.3%), 14 retinoblastomas (5.2%), and 14 other cancers (5.2%). Among these 268 patients, 54.9% (147 cases) had received radiotherapy for prior malignancies; secondary osteosarcomas occurred within the prior radiation field in 104 patients (38.8%) and outside the radiation field in 43 patients. The median latency interval between the first primary malignancy and the diagnosis of secondary osteosarcoma was 98.5 months (2–501 months). The mean follow-up times were 90.1 and 39.3 months in the primary and secondary osteosarcoma cohorts, respectively. As primary osteosarcoma mostly occurred in children and adolescents, the prognosis of this was good. This could be the main reason for substantial variation of the follow-up period.

**Table 1:** Demographics and clinicopathologic characteristics of primary and secondary osteosarcomas.

		Primary Osteosarcoma	Secondary Osteosarcoma	*p value*
**Total cases**		5860 (100)	268 (100)	
**Age at diagnosis (years)**		29.8 ± 21.5	55.1 ± 24.3	< 0.001
**Sex**				0.007
Male		3,236 (55.2)	125 (46.6)	
Female		2624 (44.8)	143 (53.4)	
**Race**				0.716
White		4447 (75.9)	206 (76.9)	
Others		1413 (24.1)	62 (23.1)	
**Marital status at diagnosis**				< 0.001
Married		1504 (25.7)	180 (67.2)	
Un-married		4356 (74.3)	88 (32.8)	
**Year of diagnosis**				0.197
1973–1994		1501 (25.6)	59(22.0)	
1995–2015		4359 (74.4)	209(78.0)	
**Tumor location**				< 0.001
Bone		5556 (94.8)	221 (82.5)	
Extra-skeleton		304 (5.2)	47 (17.5)	
**Primary site**				< 0.001
Extremity		4407 (75.2)	80 (29.9)	
Trunk		1353 (23.1)	175 (65.2)	
Unknown		100 (1.7)	13 (4.9)	
**Histology**				< 0.001
Pagetic ostosarcoma		72 (1.2)	9 (3.4)	
Non-Pagetic ostosarcoma		1675 (28.6)	48 (17.9)	
NOS		4113 (70.2)	211 (78.7)	
**Stage**				0.024
Localized		1987 (33.9)	76 (28.4)	
Regional		2331 (39.8)	104 (38.8)	
Distant		1133 (19.3)	58 (21.6)	
Unstaged		409 (7.0)	30 (11.2)	
**Grade**				0.266
I		236 (4.0)	4 (1.5)	
II		327 (5.6)	15 (5.6)	
III		1064 (18.2)	50 (18.7)	
IV		2051 (35.0)	90 (33.6)	
Unknown		2182 (37.2)	109 (40.7)	

Data are expressed as n (%) unless otherwise specified.

### Survival in primary and secondary osteosarcoma

Median CSS was not reached in the primary osteosarcoma cohort because the survival probability was greater than 50% at the last follow-up point, while it was 65 months (95% confidence interval [CI] 20.2–109.8) in the secondary osteosarcoma cohort (*P* < 0.001). The median OS was 126 months in cases of primary osteosarcoma (95% CI 101.3–150.7) and 15 months (95% CI 11.8–18.2) in cases of secondary osteosarcoma (*P* < 0.001).

### Prognostic factors associated with CSS in patients with secondary osteosarcoma

The prognostic factors for CSS for secondary osteosarcoma are shown in Table 2. In univariable analyses, younger age at diagnosis, Caucasian ethnicity, unmarried marital status, chemotherapy for prior malignancies, later year of diagnosis, first primary malignancy other than carcinoma, extraskeletal tumor location, an extremity primary site, non-pagetic osteosarcoma histology, localized disease at presentation, surgical resection, chemotherapy and no radiation therapy for osteosarcoma were significantly associated with improved CSS. These 13 factors were submitted to multivariable analysis. The results showed that age, race, year of diagnosis, a skeletal/extraskeletal tumor location, stage and surgical resection retained significance in the multivariate analysis.

**Table 2:** Univariable and multivariable analysis of each factor’s ability in predicting CSS and OS of secondary osteosarcomas.

Characteristic	CSS				OS			
	Univariable analysis		Multivariable analysis		Univariable analysis		Multivariable analysis	
	HR (95% CI)	*p* value	HR (95% CI)	*p* value	HR (95% CI)	*p* value	HR (95% CI)	*p* value
**Age at diagnosis**	1.025 (1.015–1.034)	< 0.001	1.035 (1.019–1.051)	< 0.001	1.024 (1.017–1.03)	< 0.001	1.028 (1.017–1.039)	< 0.001
**Race**								
White	1 (reference)	1 (reference)	1 (reference)					
Others	1.65 (1.072–2.540)	0.023	1.708 (1.063–2.742)	0.027	1.255 (0.916–1.720)	0.158		
**Sex**								
Male	1 (reference)				1 (reference)			
Female	0.840 (0.565–1.248)	0.388				1 (0.762–1.313)	1.000	
**Marital status**								
Married	1 (reference)	1 (reference)	1 (reference)	1 (reference)				
Un-married	0.462 (0.295–0.723)	< 0.001	1.417 (0.740–2.715)	0.293	0.435 (0.319–0.593)	< 0.001	1.136 (0.724–1.782)	0.578
**Year of diagnosis**								
1973–1994	2.461 (1.63–3.714)	< 0.001	2.644 (1.492–4.686)	< 0.001	1.461 (1.07–1.995)	0.017	1.680 (1.116–2.528)	0.0129
1995–2015	1 (reference)	1 (reference)	1 (reference)	1 (reference)				
**First primary malignancy**								
Carcinomas	1 (reference)	1 (reference)	1 (reference)	1 (reference)				
Lymphomas/leukemias	0.672 (0.393–1.152)	0.148	0.921 (0.490–1.731)	0.798	0.626 (0.427–0.920)	0.017	1.133 (0.723–1.776)	0.585
Sarcomas	0.315 (0.151–0.659)	0.002	0.592 (0.256–1.370)	0.221	0.505 (0.333–0.767)	0.001	0.913 (0.558–1.494)	0.717
Others	0.449 (0.223–0.905)	0.025	1.404 (0.589–3.346)	0.444	0.421 (0.256–0.691)	< 0.001	1.021 (0.572–1.823)	0.945
**Radiation for prior malignancies**								
Yes	1 (reference)			1 (reference)				
No	1.102 (0.740–1.64)	0.633			1.161 (0.883–1.526)	0.285		
**Chemotherapy for prior malignancies**								
Yes	1 (reference)	1 (reference)	1 (reference)	1 (reference)				
No/Unknown	1.456 (0.954–2.222)	0.081	1.157 (0.688–1.945)	0.582	1.321 (0.993–1.756)	0.056	1.067 (0.748–1.524)	0.719
**Number of primary malignancies**								
1	1 (reference)			1 (reference)				
≥ 2	1.472 (0.819–2.648)	0.196			1.411 (0.933–2.133)	0.103		
**Latency interval**	1 (0.999–1.003)	0.868			0.999 (0.998–1.001)	0.737		
**Tumor location**								
Bone	1 (reference)	1 (reference)	1 (reference)					
Extra-skeleton	0.086 (0.021–0.348)	< 0.001	0.096 (0.023–0.406)	0.001	0.734 (0.503–1.071)	0.109		
**Primary site**								
Extremity	1 (reference)	1 (reference)	1 (reference)					
Trunk	1.146 (0.736–1.785)	0.546	1.027 (0.621–1.699)	0.916	1.176 (0.870–1.590)	0.292		
Unknown	2.264 (1.034–4.957)	0.041	1.143 (0.456–2.863)	0.776	1.608 (0.866–2.986)	0.133		
**Osteosarcomas occurring within the prior radiation field**								
No/Unknown	1 (reference)			1 (reference)	1 (reference)			
Yes	1.076 (0.609–1.903)	0.801			1.501 (0.986–2.285)	0.058	1.852 (1.124–3.052)	0.016
No first radiation	1.156 (0.668–1.999)	0.604			1.534 (1.018–2.313)	0.041	1.386 (0.874–2.197)	0.165
**Histology**								
Non-Pagetic osteosarcoma	1 (reference)	1 (reference)	1 (reference)	1 (reference)				
Pagetic osteosarcoma	4.704 (1.603–13.800)	0.005	2.660 (0.828–8.551)	0.100	2.587 (1.238–5.406)	0.012	1.680 (0.756–3.734)	0.203
NOS	2.419 (1.253–4.668)	0.008	1.826 (0.908–3.670)	0.091	1.425 (0.984–2.064)	0.061	1.088 (0.727–1.630)	0.681
**Stage**								
Localized	1 (reference)	1 (reference)	1 (reference)	1 (reference)				
Regional	2.486 (1.398–4.421)	0.002	3.292 (1.701–6.370)	< 0.001	1.437 (1.008–2.048)	0.045	1.902 (1.283–2.819)	0.001
Distant	5.919 (3.145–11.138)	< 0.001	5.977 (2.930–12.191)	< 0.001	3.696 (2.476–5.517)	< 0.001	4.370 (2.775–6.881)	< 0.001
Unstaged	1.971 (0.894–4.345)	0.092	1.190 (0.477–2.972)	0.709	1.539 (0.953–2.484)	0.078	1.146 (0.663–1.979)	0.626
**Grade**								
I	1 (reference)				1 (reference)			
II	1.019 (0.106–9.800)	0.987				1.505 (0.325–6.97)	0.602	
III	1.857 (0.248–13.920)	0.547				1.941 (0.468–8.054)	0.361	
IV	2.195 (0.299–16.09)	0.439				2.397 (0.587–9.792)	0.223	
Unknown	2.346 (0.323–17.05)	0.399				2.386 (0.587–9.696)	0.224	
**Surgery**								
Yes	1 (reference)	1 (reference)	1 (reference)	1 (reference)				
No	2.85 (1.741–4.663)	< 0.001	2.346 (1.351–4.075)	0.002	2.417 (1.750–3.339)	< 0.001	1.947 (1.343–2.824)	< 0.001
Unknown	2.298 (1.421–3.716)	< 0.001	1.563 (0.782–3.124)	0.207	1.346 (0.954–1.898)	0.090	1.061 (0.654–1.722)	0.811
**Radiation**								
Yes	1 (reference)	1 (reference)	1 (reference)	1 (reference)				
No	0.567 (0.364–0.882)	0.012	1.261 (0.664–2.394)	0.478	0.610 (0.447–0.832)	0.002	1.127 (0.758–1.676)	0.553
**Radiation sequence with surgery**								
Radiation prior to surgery	1 (reference)				1 (reference)			
Radiation after surgery	2.313 (0.301–17.8)	0.421				2.285 (0.540–9.672)	0.262	
No radiation and/or cancer-directed surgery	1.939 (0.270–13.93)	0.51				2.036 (0.505–8.208)	0.317	
**Chemotherapy**								
Yes	1 (reference)	1 (reference)	1 (reference)	1 (reference)				
No/unknown	1.672 (1.122–2.491)	0.012	1.363 (0.818–2.271)	0.234	1.631 (1.24–2.145)	< 0.001	1.345 (0.958–1.887)	0.087

### Independent prognostic factors associated with OS in patients with secondary osteosarcoma

**Figure 1:**
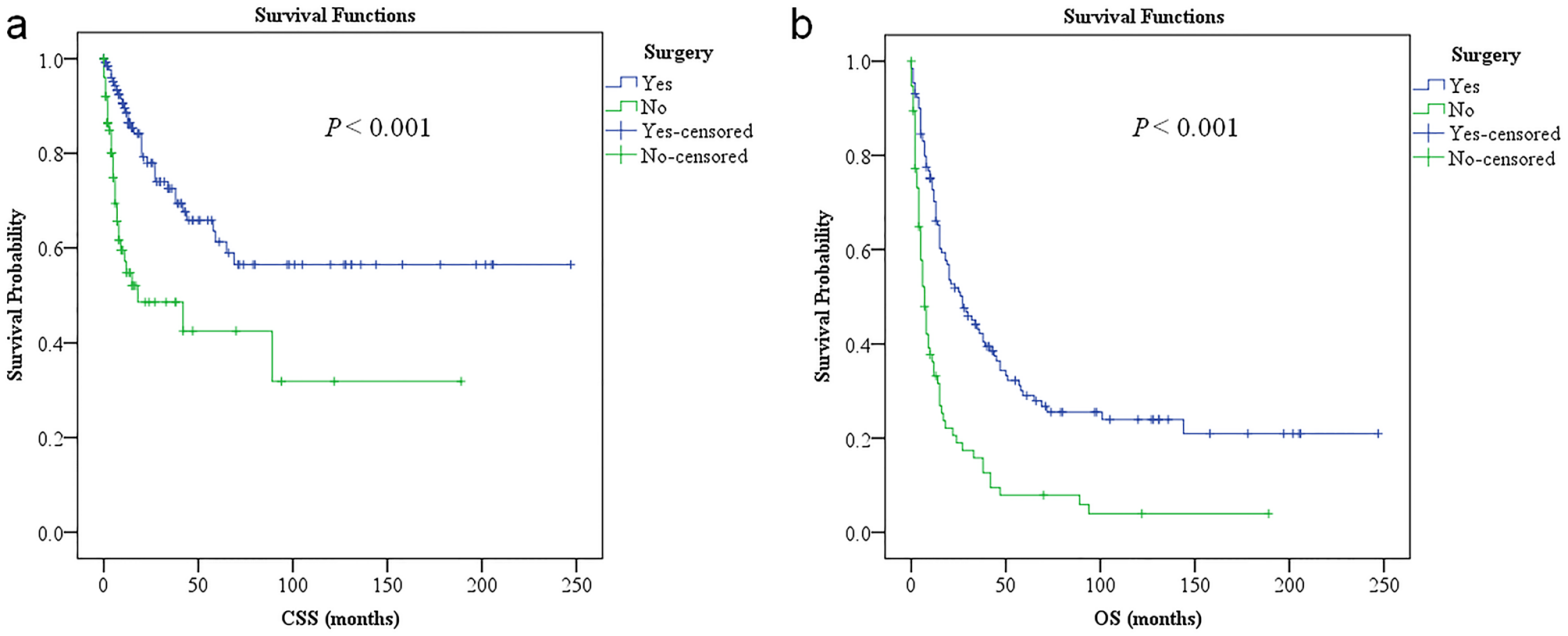
Patients who underwent surgical resection for secondary osteosarcomas had longer CCS (**a**) and OS (**b**) compared with patients who didn’t. CSS cancer-specific survival, OS overall survival.

Univariable analysis suggested that younger age at diagnosis, unmarried marital status, later year of diagnosis, first primary malignancies other than carcinomas, chemotherapy for prior malignancies, osteosarcomas occurring outside the prior radiation field, non-Pagetic osteosarcoma histology, localized disease at presentation, surgical resection, chemotherapy and no radiation therapy for secondary osteosarcoma were favorable predictors of OS. Similar to CSS, age, year of diagnosis, stage, and surgical resection for osteosarcoma were independent prognostic factors associated with OS in multivariable analyses, but with the addition of osteosarcoma occurring within/outside the prior radiation field. To be noted, surgical resection was an independent favorable factor for both CSS and OS in the present cohort (Fig. 1). Unlike CSS, however, race and skeletal/extraskeletal tumor location did not have any bearing on OS among patients with secondary osteosarcoma (Table 2).

### Building and validating a prognostic nomogram for CSS in patients with secondary osteosarcoma

**Figure 2:**
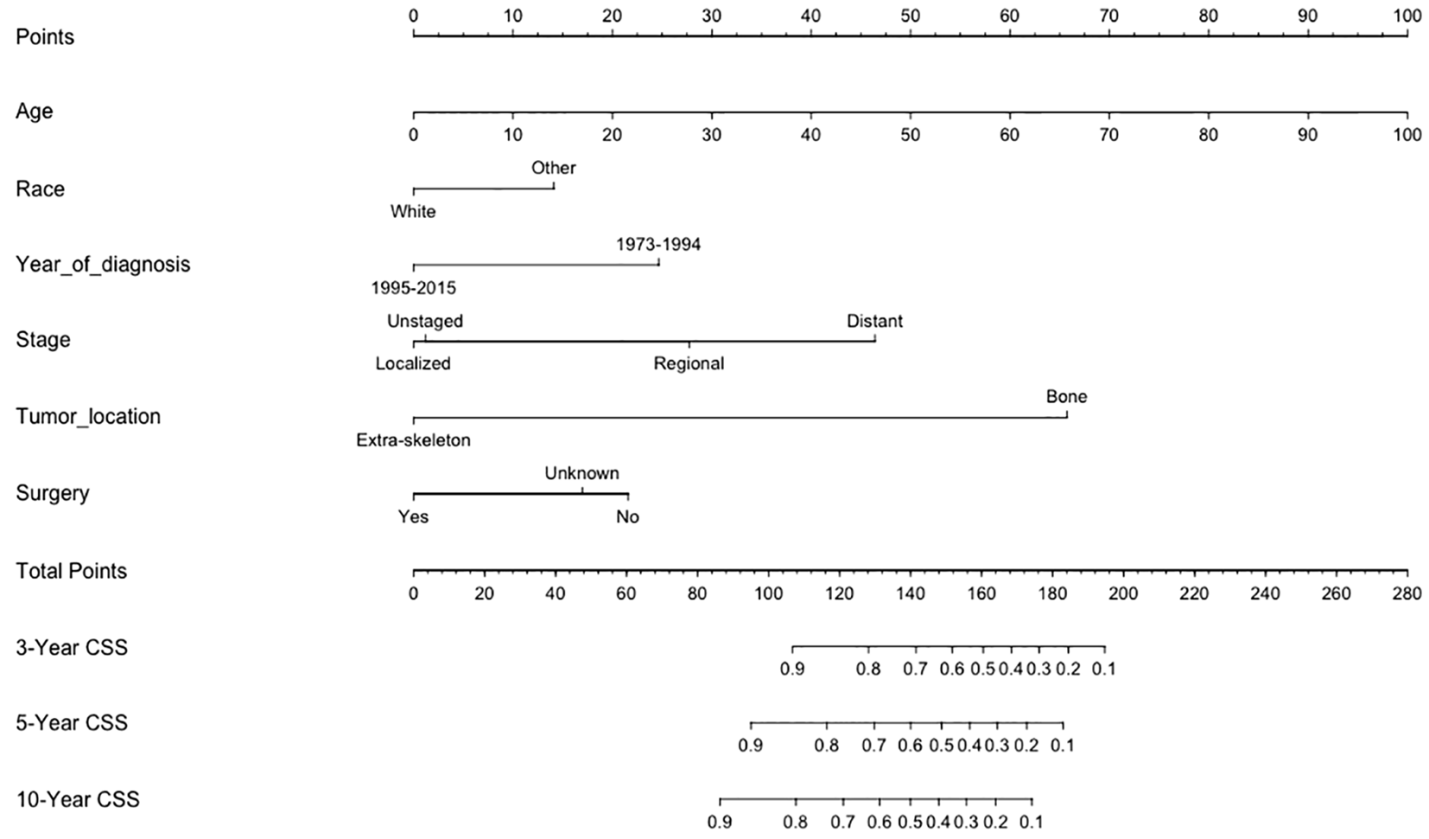
Nomogram predicting 3-year, 5-year and 10-year cancer-specific survival (CSS) of patients with secondary osteosarcomas. The nomogram summed the points identified on the scale for each variable. The total points projected on the button scale indicate the probabilities of 3-year, 5-year and 10-year CSS. CSS cancer-specific survival.

The nomogram for predicting CSS among patients with secondary osteosarcoma was formulated using the significant independent factors, including age, race, year of diagnosis, skeletal/extraskeletal tumor location, stage, and surgical resection. The nomogram showed that the largest contributions to prognosis were the location (skeletal or extraskeletal tumor) and age at diagnosis, followed by stage and year of diagnosis. Each variable was assigned a score according to the demographic and clinical features of individual patient (Table 3). By adding up these scores according to a patient’s condition, the total score was computed by summing the individual scores. Then, the total score was located on the total point line, and a straight line could be drawn to estimate the patient’s probability of 3-year, 5-year, and 10-year CSS from the nomogram (Fig. 2).

**Table 3:** Score assignment for each variable included in the nomogram.

Variables	Points
**Age at diagnosis**	
0	0
10	10
20	20
30	30
40	40
50	50
60	60
70	70
80	80
90	90
100	100
**Race**	
White	0
Other	14
**Year of diagnosis**	
1995–2015	0
1973–1994	25
**Stage**	
Localized	0
Regional	28
Distant	46
Unstaged	1
**Tumor location**	
Bone	66
Extra-skeleton	0
**Surgery**	
Yes	0
No	22
Unknown	17

**Figure 3:**
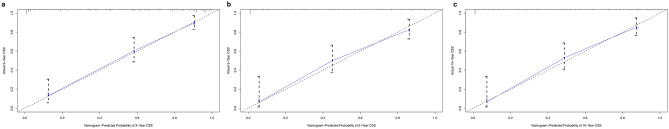
Calibration curves of the nomogram for predicting 3-year CSS (**a**), 5-year CSS (**b**) and 10- year CSS (**c**). CSS cancer-specific survival.

The C-index for the CSS prediction nomogram was 0.826 (95% CI: 0.787–0.865) for the training cohort and was confirmed to be 0.791 through bootstrapping validation, which suggested that the model had good discriminative ability. The calibration plots for CSS probability at 3-year, 5-year, and 10-year showed that the concordance between predicted and observed survival was optimal (Fig. 3).

## Discussion

The incidence of primary osteosarcoma has always been considered higher in males than in females^25^, while the current study revealed that in cases of secondary osteosarcoma, the majority of the patients were females. The proportions of patients with different races were consistent for primary and secondary osteosarcomas. This study showed that amongst patients with secondary osteosarcomas, 72.4% were older than 40 years at the time of diagnosis, the result was similar to previous reports^16,26^. Primary osteosarcoma mostly occurred in the long bones of the extremities near the metaphyseal growth plates^25^. However, we observed that secondary osteosarcomas were more likely to be located at non-extremity sites. In the current study, the authors found that the most common primary malignancies were carcinomas, followed by sarcomas and lymphomas/leukemias. These results were inconsistent with previous studies^27,28^. Distant metastases were present at diagnosis in 21.6% of secondary osteosarcoma patients. Radiation is a well-documented etiological factor of osteosarcoma, with the median interval between radiation and the occurrence of osteosarcoma reported to be 12–16 years^25^. This study observed a shorter post-radiation latency because the median latency interval between the diagnosis of first primary malignancies and osteosarcoma was 98.5 months, as the exact date of prior radiotherapy was not available. In this cohort, the pelvis was the most commonly affected site, 38.8% of secondary osteosarcomas occurred within the prior radiation field.

Most previous SEER studies on osteosarcoma either treated primary and secondary osteosarcoma together or were limited to osteosarcoma of specific histological subtypes^29–32^. There was only one study focusing on secondary osteosarcoma from SEER data, published nearly 20 years ago, which included only 133 patients and indicated that secondary osteosarcoma had poorer OS than primary osteosarcoma. However, that study did not evaluate CSS nor analyze the impact of any treatment on survival^14^. A later study reported that radiation-induced secondary osteosarcoma proved to have similar outcomes to primary osteosarcoma^33^. However, a recent study suggested that the prognosis of secondary osteosarcoma may be more favorable than that of primary osteosarcoma^34^. The survival and prognostic factors of secondary osteosarcoma remain unclear. So, identifying accurate prognostic factors has clinical importance for guiding personalized cancer therapy. The present study provides detailed survival data, and it could be the largest cohort study on secondary osteosarcoma reported to date. Furthermore, an optimal graphical validated nomogram was developed for predicting CSS. The nomogram model exhibited high discriminative accuracy in the training cohort (C-index = 0.826), which was further confirmed in the internal validation cohort (C-index = 0.791). This study suggests the excellent performance of this nomogram for estimating the prognosis of secondary osteosarcoma, as the calibration plots confirmed good concordance for the prediction of CSS at 3-, 5-, and 10-years. To the best of our knowledge, this is the first prognostic nomogram developed for secondary osteosarcoma.

This study revealed that patients with secondary osteosarcoma had poorer CSS and OS than patients with primary osteosarcoma. We identified age, race, year of diagnosis, skeletal/extraskeletal tumor location, stage, and surgical resection as independent factors for CSS. For OS, the independent prognostic factors included age, year of diagnosis, stage, surgical resection, and osteosarcoma occurring within/outside the prior radiation field. Notably, patients with secondary osteosarcomas occurring within the irradiated field had inferior OS compared to patients with secondary osteosarcomas occurring outside the irradiated field. CSS was similar between groups. This suggests that the difference in OS was caused by factors other than secondary osteosarcoma itself.

Previous studies have reported that surgical resection significantly improves disease-free survival and OS in patients with secondary osteosarcoma^28,33^. We also observed significant differences in CSS and OS between secondary osteosarcoma cases with or without surgical resection, which demonstrated that surgical resection was an independent factor significantly improving CSS and OS in the present cohort. For osteosarcomas occurring within the prior radiation field, one important issue that must be addressed is that radiation therapy can prolong postoperative complications because the condition of the operative field is entirely altered after radiotherapy^33^. For these patients, surgical options should be prudently adopted^35^. In the present study, data on the postoperative complications were not available due to the limitations of the SEER database; however, the favorable CSS and OS findings strongly justify the surgical resection of secondary osteosarcoma.

Intensive chemotherapy has considerably improved the prognosis of patients with primary osteosarcoma^9^. For secondary osteosarcoma, Shaheen et al. reported that patients treated aggressively with a combination of chemotherapy and surgical resection had better outcomes than patients treated with surgical resection alone^33^. The present study did not demonstrate significant benefits of chemotherapy on CSS or OS in multivariable analyses. However, the heterogeneous regimens and intensity of chemotherapy over more than 40 years may have limited the statistical power of this study. Prior myelosuppressive chemotherapy and/or radiotherapy may limit the tolerance of patients with secondary osteosarcoma who undergo subsequent intensive chemotherapy, and we strongly recommend the prophylactic use of myeloid growth factors after chemotherapy^36^.

This study had several limitations. First, due to the retrospective study design, selection bias was unavoidable. Second, the SEER dataset lacks data on doses of radiotherapy or chemotherapy regimens, and we were therefore unable to evaluate the impacts of these factors on the development and survival of secondary osteosarcoma. Third, due to the rarity of this disease, we were not able to validate the constructed nomogram using other cohorts.

## Conclusion

We developed a practical nomogram that provided individual predictions of CSS for patients with secondary osteosarcoma using five clinicopathological factors and one treatment-related factor. Bootstrapping validation of the model confirmed its good performance. This nomogram may help clinicians with prognostic evaluations and with the development of individualized therapy for this aggressive disease. Future prospective studies are required to further determine the impacts of different treatment modalities on the survival of patients with secondary osteosarcoma.

## Data Availability

Data for this manuscript are available after formal request to the corresponding authors.
